# Agronomic treatments to avoid presence of seeds in Nadorcott mandarin II. Effect on seed number per fruit and yield

**DOI:** 10.1371/journal.pone.0278934

**Published:** 2022-12-09

**Authors:** Alfonso Garmendia, María Dolores Raigón, Francisco García-Breijo, José Reig, Roberto Beltrán, Carlos Zornoza, Nuria Cebrián, Hugo Merle

**Affiliations:** 1 Instituto Agroforestal Mediterráneo, Universitat Politècnica de València, Valencia, Spain; 2 Instituto de Conservación y Mejora de la Agrobiodiversidad Valenciana/Departamento de Química, Universitat Politècnica de València, Valencia, Spain; 3 Departamento de Ecosistemas Agroforestales, Universitat Politècnica de València, Valencia, Spain; 4 Instituto Cavanilles de Biodiversidad y Biología Evolutiva, Universidad de Valencia, Valencia, Spain; 5 S.A. Explotaciones Agrícolas Serrano (SAEAS), Valencia, Spain; United States Department of Agriculture, UNITED STATES

## Abstract

Nadorcott is a well-established and appreciated mandarin by the fresh market. However, it produces seeds due to cross-pollination with other compatible varieties, which is quite frequent in most producing countries. Consumers prefer seedless mandarins and, therefore, citrus growers need techniques to avoid seeds forming. This study aims to evaluate the effect of six treatments (ammonium nitrate, potassium nitrate, sulfur, saccharose, methylcellulose, callose) on seed number per fruit when applied to Nadorcott trees. In this way, we evaluate which of them is more efficient and can be used in the future as an agronomic treatment to avoid seeds in mandarins. The effect of treatments on yield and fruit quality is also reported. To fulfill this main objective, a randomized complete block design experiment with three applications at flowering was performed on trees. Of the six tested treatments, only elemental sulfur was able to significantly reduce seed number by 87% compared to the positive control. This is a very novel result because it is the first time that such an effective treatment has been found. The biggest seed number per fruit was obtained for the saccharose treatment. Treatments did not significantly influence yield or fruit quality. These results are entirely consistent with a previous study that evaluated the effect of the same products on pollen tube growth, and they can help to develop new techniques. Nevertheless, more studies are necessary to test, for example, different treatment doses.

## Introduction

Nadorcott is a highly appreciated mandarin by the fresh market. It is well-established in many citrus-producing countries like Spain, South Africa, USA, Peru, etc., with high yields and good profitability [1]. This variety, also known as ’Afourer’, derives from ‘Murcott’ (*Citrus reticulata* Blanco x *C*. *sinensis* (L.) Osbeck). It produces seeds due to cross-pollination with other compatible varieties (e.g., cv. Oronules and cv. Nova) [2, 3]. Most cultivated mandarin varieties are self-incompatible and, thus, produce seedless fruit in the absence of cross-pollination [4]. However, when mandarin varieties are grown close to compatible varieties, they produce several seeds per fruit [5]. Citrus flowers are pollinated mainly by bees (*Apis mellifera* L.) with long-distance pollen dispersal [6]. Therefore, cross-pollination between mandarins is frequent due to the variety of mosaics and pollinators’ work [7].

Most consumers clearly prefer seedless fruit. In fact new seedless varieties, or traditional seedy varieties grown in isolation from other varieties (without cross-pollination), often fetch better prices on markets [8]. However, the establishment and expansion of new seedless citrus varieties are not widespread for several reasons: adaptation problems to climate conditions, poor fruit-set and yield, inappropriate organoleptic characteristics, etc. [9, 10]. Consequently, in producing countries, most cultivated varieties are traditional seedy varieties. For example in Spain, more than 90% of grown mandarins are seedy varieties [11]. Therefore, many farmers need treatments to avoid seeds appearing in these traditional varieties [12].

Nowadays, available techniques to reduce seed number per fruit in traditional mandarins are ineffective or too expensive [12, 13]. The most effective horticultural technique followed to reduce seed number per fruit is net covering [12, 14]. The net covering technique consists of covering trees with an anti-insect net to prevent pollinators reaching flowers to, thus, avoid pollination and fertilization. However, this technique is very costly and recent studies have shown that it can reduce yields [12, 14]. Insect repellents, plant growth regulators, copper, and any combination of the above, have been tested, but prove poorly effective [12, 15, 16]. In fact none of these products are currently used by citrus farmers to reduce seed number because of their poor effectiveness, which is insufficient for market requirements.

For all these reasons, we started our research work to find new treatments capable of reducing seed number in traditional seedy varieties, more specifically in Nadorcott mandarin given its marked interest. In a first phase, the effect of seven products on pollen tube growth on Nadorcott stigmas was evaluated by the research team [13]. The results showed that the elemental sulfur treatment inhibited pollen tube growth by 94–100%. The saccharose treatment seemed to stimulate pollen tube growth, but with no statistically significant differences with the positive control [13]. These are very promising laboratory results because previous attempts have not been effective. Mesejo [16] proposed using gibberellic acid at different concentrations to reduce seed number [12, 17, 18]. Similarly, copper sulfate (CuSO_4_ • 5H_2_O) [15], and products based on *Capsicum annuum* L. and zinc, have been initially proposed, but their effectiveness is poor [12, 19, 20]. Therefore, the next phase for our research team was to test these promising laboratory results [13] under field conditions. For this second phase, the research questions were: (i) to what extent do these products reduce seed number when applied to whole trees?; (ii) do these treatments affect fruit yield or quality? By answering these questions, we can assess which of the treatments is more effective under field conditions and can be used to develop an agronomic treatment that prevents seeds in mandarins. In addition, the effects of treatments on yield per tree, fruit quality and secondary effects, such as phytotoxicity, were evaluated. To fulfill this objective, a complete experiment with six replicates and eight treatments (including positive and negative controls) was carried out on entire trees under field conditions. For decades, researchers and agronomists have unsuccessfully searched for a useful treatment to prevent seeds. A highly effective novel result is herein reported.

## Materials and methods

### Experimental site and plant material

The experiment was conducted in a private commercial orchard in Montserrat, the Valencia province, east Spain (39º 21’ 35” N 0º 32’ 44” W; altitude 150 m) in April 2017. The orchard belongs to SAEAS (Sociedad Anónima Explotaciones Agrícolas Serrano). By means of agreements, the research team frequently collaborates between SAEAS and the Polytechnic University of Valencia. As SAEAS approved field site access, no additional permits were required. The general site climate was Mediterranean oceanic, with long-term average annual rainfall of 450 mm and an average annual air temperature of 19 ºC (weather station at 39º 22’ N 0º 27’ W). Soil was calcareous sandy clay loam with a pH of 8.06 and 5.2% limestone. The plot soil was homogeneous throughout the site.

Nine-year-old Nadorcott mandarin trees budded onto *Citrus macrophylla* Wester rootstock were used. Trees with drip irrigation were stood 6 x 4 m apart. The age and irrigation of all the treated trees in the experiment were the same. Orchard management was carried out under standard cultural conditions with no other treatments during the flowering period, except for the experimental treatments. The experimental plot covered a total surface area of 250 m^2^ and was close (less than 100 m) to other pollen-compatible mandarin plots (cv. ‘Clemenules’ and cv. ‘Nova’) that favor cross-pollination.

### Treatments

In this experiment, treatments corresponded to laboratory-tested products from a previous study carried out by the research team [13]. Products were initially selected to modify the physico-chemical characteristics of stigma secretion, such as viscosity or pH, of Nadorcott flowers to, thus, prevent pollen tube growth. We selected products from two main groups: saccharides/polysaccharides and inorganic salts.

Specifically, eight treatments were included in this experiment to be applied to whole trees: a positive control (C+, untreated trees exposed to cross-pollination), a negative control (C-, trees covered with anti-insect nets to avoid pollination), three inorganic fertilizers (ammonium nitrate, potassium nitrate, sulfur) and three saccharides (saccharose, methylcellulose, callose). A randomized complete block design was used with six replicates per treatment distributed in three plots (A, B, C). Each plot (A,B, C) was divided into two blocks: A (blocks 1 and 2), B (blocks 3 and 4) and C (blocks 5 and 6) (S1 Fig). That is, each treatment was applied to six Nadorcott trees according to the experimental design (S1 Fig). Each block pair was a different plot separated by a small wall, which could influence, for example, the action of pollinators (blocks 1 and 2 were further outside than blocks 3 and 4 and were, therefore, closer to the plots with compatible varieties). All the 48 treated trees were surrounded by eight untreated trees as edges to minimize any possible interactions between treatments (the white squares in S1 Fig).

For the negative control (C-), trees were covered with anti-insect nets before flowering (March) and nets were left on trees until the end of the flowering period (May). For the positive control (C+), trees were neither covered with nets nor treated with products, but were only labeled and exposed to natural open pollination during the flowering period (the lots with compatible mandarin varieties were close to the experimental plot). The other treatments were applied with a hand duster powder sprayer (Matabi, 1.5 L). These treatments were applied 3 times throughout the flowering period. The exact application dates were April 11, with 30% of flowers at anthesis (full bloom), and then every 7 days on April 18 and April 25. For each treatment and application date, 50 g of product per tree were used.

### Sampling and measurements

Several weeks after treatments, trees were observed to search for phytotoxic side effects. On May 3 and May 29, a visual assessment of leaf defoliation, chlorosis and necrosis was made. In September, when fruit were sufficiently big, the seed number per fruit was counted. For this purpose, 20 fruit per tree (120 fruit per treatment) were sampled. Fruits were cut in half and the seed number in each fruit was counted. For the ammonium nitrate treatment, only 105 fruit were sampled because this treatment had a negative effect on fruit set and there were not enough fruit available for several trees. By including all the treatments, 945 fruit were cut to count their seed number.

When fruit had ripened (10 months after treatments, February 2018), trees were harvested and the yield per tree was noted. At this time, the equatorial diameter (mm) of 80–90 fruit per treatment was measured by an electronic digital slide gauge (model CD-15 DC; Mitutoyo (UK) Ltd., Telford, UK) to within 0.01 mm accuracy. Four fruit per tree (24 fruit per treatment) were sampled for the fruit quality analysis. The peel color measurement in the CieLab space was taken at two random points on citrus fruit skin using a colorimeter (Konica Minolta CR-300, Photo Imaging Inc., Mahwah, NJ, USA) to obtain color components (L, a, and b). The color index (CI) was calculated using the equation CI = 1000*a/(L*b), where “L” indicates brightness, “a” denotes chromaticity on a green (−) to a red (+) axis, and “b” depicts chromaticity on a blue (−) to a yellow (+) axis. This index is widely used in the citrus industry as a ripening index.

The four fruit per tree were weighed and squeezed together to calculate the proportion of pulp, juice and skin by being weighed on an analytical balance (Cobos, ± 0.001 g accuracy). Juice was filtered and used to determine the total soluble solids (TSS) content (TSS, ºBrix; method 932.12), pH (method 981.12), total acidity (TA) and % anhydrous citric acid (CA) (method 942.15) according to the Association of Official Analytical Chemists [21]. Additionally, the TSS/CA ratio was calculated to define citrus fruit ripeness or the maturity index (MI). The vitamin C concentration in citrus (mg/100 g juice) was determined by potentiometric titration with chloramine T using an automatic titration apparatus (702 SM Titrino, Metrohm, Herisau, Switzerland).

### Statistical analysis

For each dependent variable (seed number per fruit, yield and the quality of the fruit variables), the mean, median, standard deviation and standard error per treatment were calculated. The block effect was initially analyzed for each variable. When significant differences were found among blocks for a variable, this effect was taken into account for the subsequent analyses. The Shapiro-Wilk test was used to check for normality of residuals, while the Levene’s test was applied to analyze the homogeneity of group variances.

For the comparison of means, when the requirements for homoscedasticity and normality of residuals were met, an ANOVA was applied with the Tukey *post hoc* Honest Significant Differences test (HSD). When requirements were not met, a non parametric method, namely the Kruskal-Wallis rank sum test, was selected to compare the means among treatments. In these cases, a *post hoc* Nemenyi’s non parametric test [22] was used when statistically significant differences were found. For some variables, the eta-squared statistic was calculated to assess the effect size. The Fisher test [23] with Holm’s correction *post hoc* test [24] was applied to analyze the frequencies of fruit with or without seeds.

All the statistical analyses were performed using R [25] with RStudio gui [26] and some extra packages: agricolae [27], PMCMRplus [28] and ggplot2 [29]. The comprehensive data and R scripts for the statistical analysis are available as S1 File.

## Results

### Effect of treatments on seed number per fruit

Including all the treatments, 945 data on seed number per fruit were used for the statistical analysis. To check whether there was any spatial effect that interacted with the possible effect of treatments on seed number per fruit, we analyzed if there were significant differences between blocks (S2 File). The Kruskal-Wallis test for the effect of blocks on seed number showed no statistically significant differences (S2 File).

**Fig 1:**
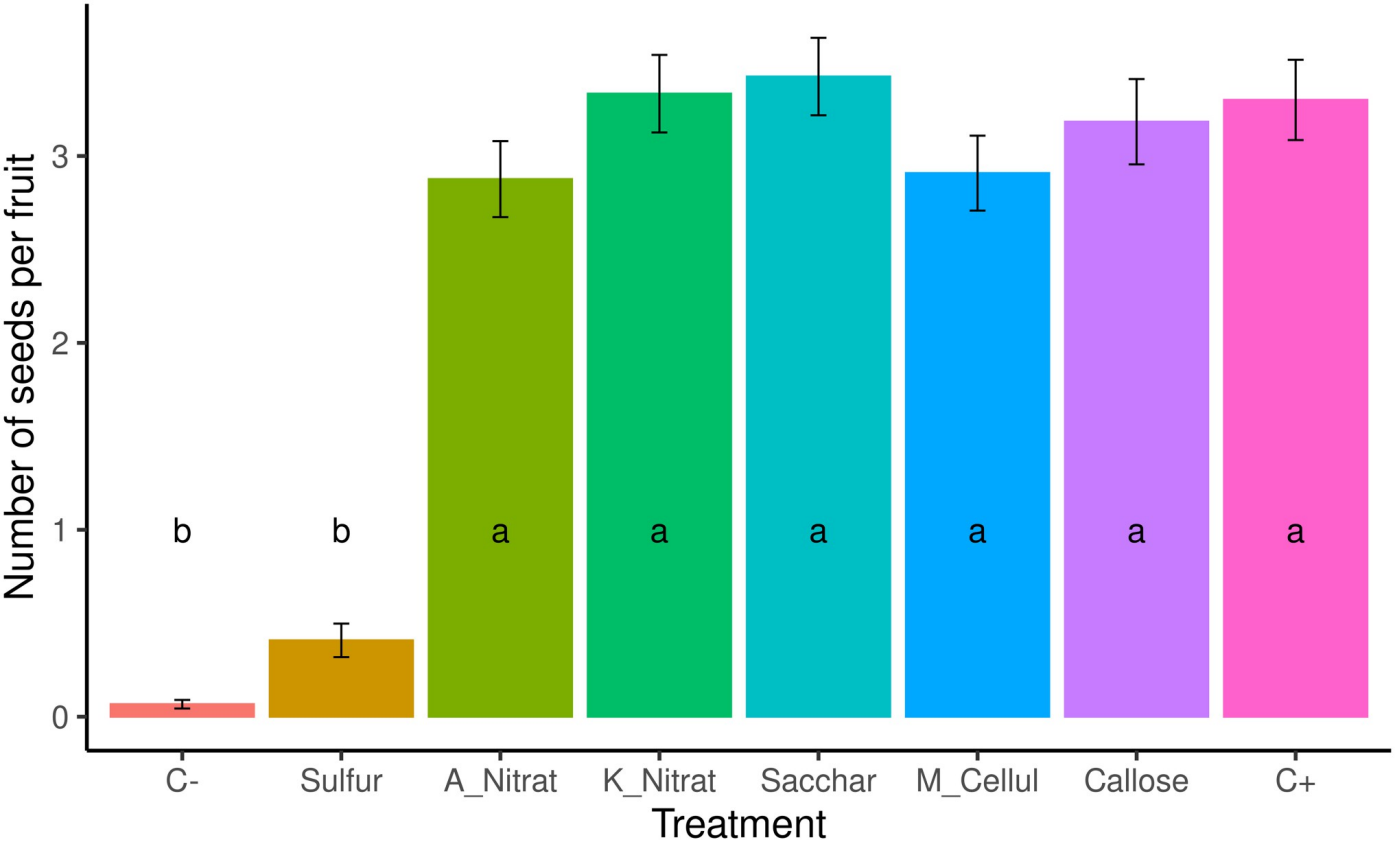
Bar plot for the effect of treatment on the average seed number per fruit. Different letters denote the statistically significant differences in the Kruskal-Wallis test (*post hoc* Nemenyi (Tukey) test), for alpha ≤0.05 (KW value = 56.133; p-value = 3.87e-67). Error lines correspond to the standard error. Treatments are noted as: C-, negative control; Sulfur; A_Nitrat, ammonium nitrate; K_Nitrat, potassium nitrate; Sacchar, saccharose; M_Cellul, methyl cellulose; Callose; C+, positive control.

Next we analyzed the effect of treatments on seed number. The Kruskal-Wallis test for the effect of treatments on seed number per fruit showed statistically significant differences among treatments (KW value = 56.133; p-value = 3.87e-67; Fig 1). The negative control (net-covered trees) had the smallest seed number (0.07±0.02, Table 1), while the positive control (open pollination) showed an average of 3.30 seed per fruit, which confirmed that cross pollination was frequent between the experimental plot and other nearby cross-compatible varieties. The elemental sulfur treatment was the only one to significantly reduce the number of seeds compared to C+ (Fig 1). No other treatment had such an effect or gave statistically significant differences with C+ (Fig 1). The saccharose treatment had the biggest seed number per fruit (3.43 seeds), but the difference with C+ was small and not statistically significant (Table 1). Sulfur reduced seed number by 87% (from 3.3 to 0.41 seeds per fruit) when the elemental sulfur treatment was pairwise-compared to C+ (S3 and S4 Files).

**Table 1:** Average seed number per fruit achieved during each treatment.

Treatment	N	mean	KW	sd	se	Shapiro
C-	120	0.07	b	0.25	0.02	7.94735E-22
Sulfur	120	0.41	b	0.98	0.09	1.10006E-18
A_Nitrat	105	2.88	a	2.08	0.20	9.64972E-05
K_Nitrat	120	3.33	a	2.27	0.21	8.36177E-05
Sacchar	120	3.43	a	2.27	0.21	0.000165486
M_Cellul	120	2.91	a	2.20	0.20	1.09471E-05
Callose	120	3.18	a	2.50	0.23	7.52994E-07
C+	120	3.30	ab	2.35	0.21	0.000123695

Table note: KW, the Kruskal-Wallis *post hoc* test was Tukey Nemenyi. Different letters mean statistically significant differences for alpha ≤ 0.05 (KW value = 56.133; p-value = 3.87e-67); sd, standard deviation; se, standard error; Shapiro, p-value for the Shapiro-Wilk normality test, p-value below 0.05 indicates non normal distribution. Shapiro test for residuals p = 4.98e-17, Levene p = 2.0275606 e-51, data did not meet normality or homoscedasticity criteria for the ANOVA analyses.

### Effect of treatments on the frequency of fruit with and without seeds

**Fig 2:**
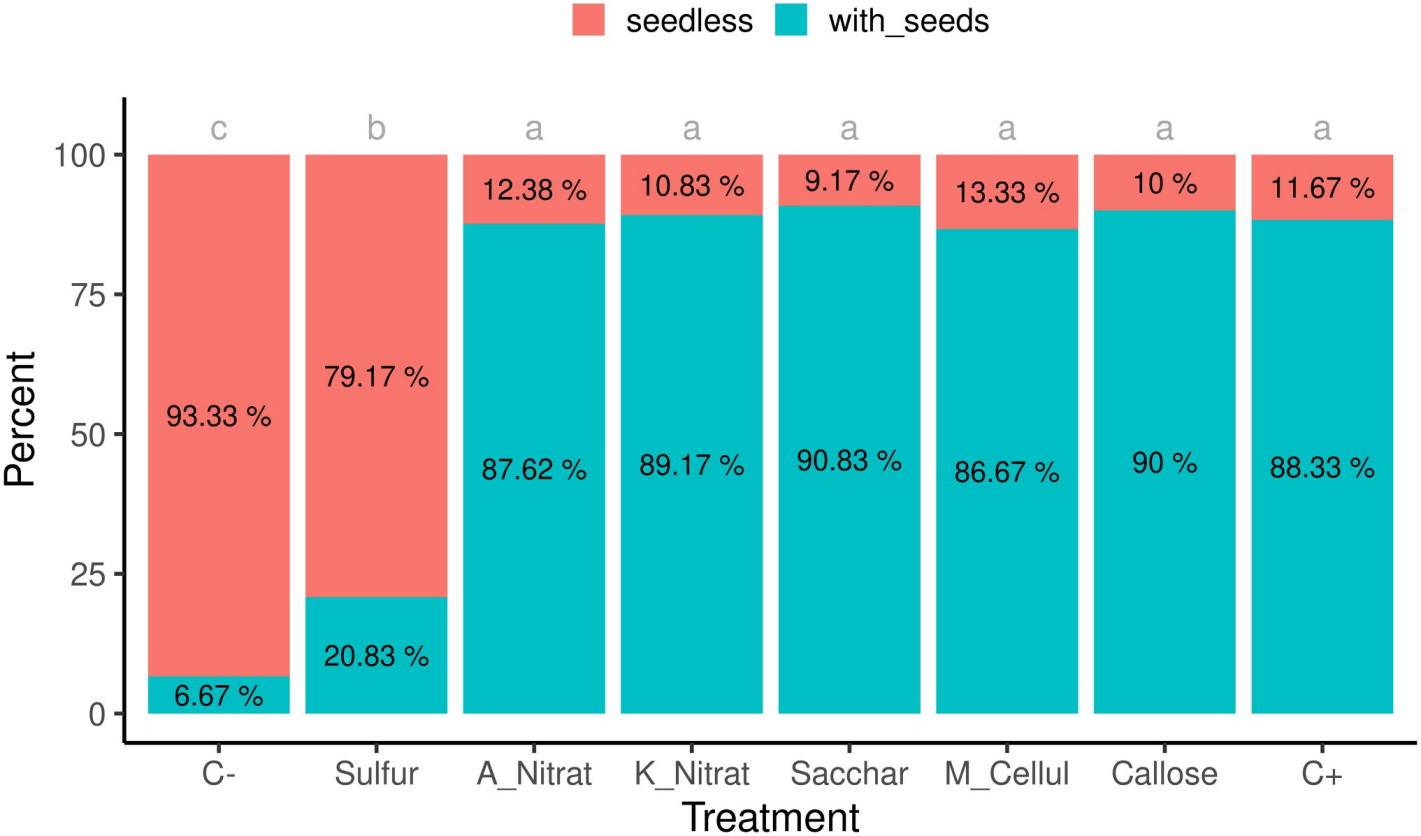
Percentage of seeded and seedless fruit per treatment. Blue (with seeds) indicates the fruit with one seed or more; red (seedless) denotes seedless fruit. Different gray letters represent statistically significant differences in the chi-square according to the Fisher test with Holm’s correction *post hoc* test (p-value = 5e-04). Treatments are noted as: C-, negative control; Sulfur; A_Nitrat, ammonium nitrate; K_Nitrat, potassium nitrate; Sacchar, saccharose; M_Cellul, methyl cellulose; Callose; C+, Positive control. The total number of fruit per treatment was 120, except for ammonium nitrate with 105.

The positive control displayed that 88.3% of fruit contained seeds, while only 6.7% were seedy fruit in the negative control (Fig 2). According to the average seed number per fruit, the sulfur treatment significantly reduced the frequency of seedy fruit compared to C+ (from 88.3% to 20.8%; p value = 5e-04). The other treatments displayed a similar seedy fruit frequency to C+ (Fig 2). When analyzing seed number per fruit, but excluding the seedless fruit, a different behavior was noted for seed number per fruit in the sulfur treatment *versus* the other treatments (S4 File). When considering only the seedy fruit, the sulfur treatment continued to partially reduce seed number (S4 File).

### Effect of treatments on yield

**Fig 3:**
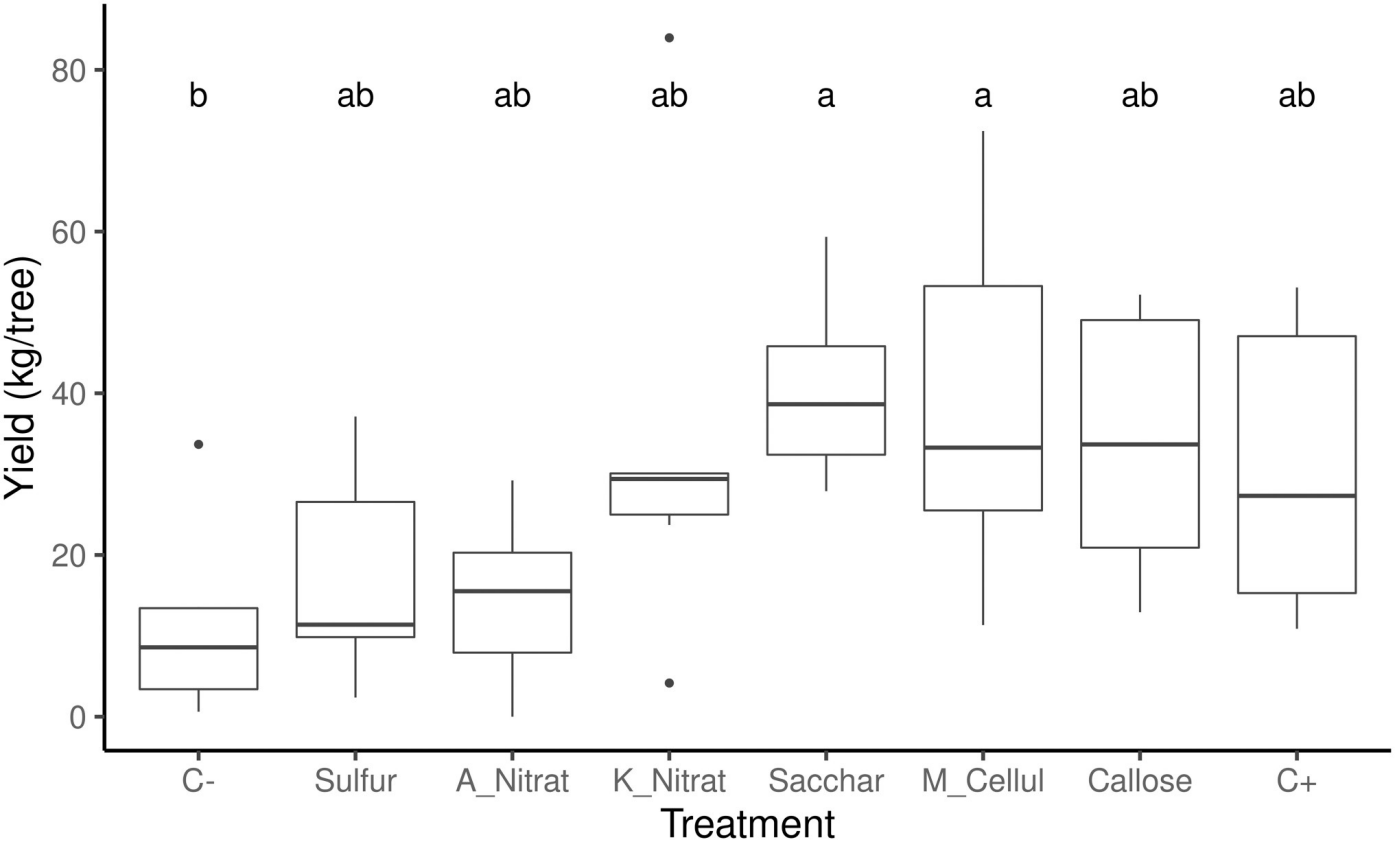
Box plot for the effect of treatments on yield. Different letters mean statistically significant differences in the ANOVA Tukey *post hoc* test (HSD) for alpha ≤ 0.05, Df = 40, p value = 0.028. Treatments are noted as: C-, negative control; Sulfur; A_Nitrat, ammonium nitrate; K_Nitrat, potassium nitrate; Sacchar, saccharose; M_Cellul, methyl cellulose; Callose; C+, Positive control.

In order to analyze the effect of treatments on yield, 48 yield data were used, one for each treated tree. When analyzing the effect of blocks on yield, statistically significant differences were found (S6 File). There was a small difference between block 2 (higher yield) and block 5 (lower yield) (S6 File). Therefore, the effect of treatments on yield was analyzed by taking into account the interaction with blocks. The ANOVA analysis for the effect of treatments on yield showed statistically significant differences among treatments (F-value = 2.56; p-value = 0.028; Fig 3). The positive control yielded an average of 30.6 kg per tree, while the negative control obtained 11.4 kg per tree. This difference was not statistically significant (Fig 3 and Table 2). The yield data displayed wide variability among treatments, with several anomalous data below 10 kg (Fig 3). Although the negative control and the elemental sulfur treatment did not show any statistically significant differences with C+, a low yield trend appeared. The saccharose treatment had the highest yield (40.5 kg) and statistically significant differences with C-.

**Table 2:** Average yield per tree achieved in each treatment.

Treatment	N	Median	Mean	sd	se	Shapiro	HSD
C-	6	8.575	11.36	12.17	4.97	0.13	b
Sulfur	6	11.38	17.20	13.70	5.59	0.23	ab
A_Nitrat	6	15.52	14.59	10.61	4.33	0.89	ab
K_Nitrat	6	29.4	33.46	26.64	10.88	0.06	ab
Sacchar	6	38.64	40.51	11.66	4.76	0.64	a
M_Cellul	6	33.275	38.86	22.73	9.28	0.76	a
Callose	6	33.67	33.88	16.93	6.91	0.31	ab
C+	6	27.315	30.58	18.86	7.70	0.16	ab

Table note: sd, standard deviation; se, standard error; Shapiro, p-value for the Shapiro-Wilk normality test, a p-value below 0.05 indicates non normal distribution; HSD, Tukey’s Honestly Significant Difference test, where different letters depict statistically significant differences in the ANOVA Tukey *post hoc* test for alpha ≤ 0.05 (F-value = 2.56; p-value = 0.028). Shapiro test for residuals = 0.0511, Levene p = 0.391, data meet normality and homoscedasticity requirements.

### Effect of treatments on fruit quality

Only a few statistically significant differences were found in the fruit quality variables. Weight, % rind, % juice, % pulp, pH, total acidity, ripeness and vitamin C did not show any statistically significant differences when comparing treatments to C+ (Table 3). The negative control had the smallest fruit diameter with statistically significant differences (57.5 C- vs. 60.73 mm C+), while the ammonium nitrate treatment showed statistically significant differences in the TSS content. In general, the seeded fruit were about 3 mm larger than the seedless fruit (S7 File). Finally, the saccharose treatment obtained a higher CI (12.4) with statistically significant differences, but the effect size measured by eta-squared was small (η^2^ = 0.075). The elemental sulfur treatment did not show any statistically significant differences compared to C+ (Table 3).

**Table 3:** Effect of treatment on the fruit quality variables.

Treatment		C-	Sulfur	A_Nitrat	K_Nitrat	Sacchar	M_Cellul	Callose	C+
**Diameter**	Mean	57.48	61.28	59.55	60.12	61.47	59.97	61.59	60.73
	KW	d*	a	c	abc	ab	bc	a	abc
**Weight**	Mean	88.07	96.98	89.18	95.5	93.02	92.13	91.98	92.48
	HSD	a	a	a	a	a	a	a	a
**CI**	Mean	8.23	8.84	8.93	8.37	12.36	8.93	8.55	8.64
	KW	c	bc	b	bc	a*	bc	bc	bc
**% Rind**	Mean	26.88	27.76	27.32	26.35	29.00	26.83	28.09	27.50
	HSD	a	a	a	a	a	a	a	a
**% Juice**	Mean	49.26	45.96	46.88	48.67	44.51	48.35	43.97	49.06
	HSD	a	a	a	a	a	a	a	a
**% Pulp**	Mean	23.87	26.29	25.80	24.99	26.43	24.82	27.94	23.44
	HSD	a	a	a	a	a	a	a	a
**pH**	Mean	2.82	2.85	2.79	2.84	2.82	2.85	2.83	2.80
	KW	ab	ab	b	ab	ab	a	ab	ab
**Soluble solids**	Mean	12.50	12.47	13.24	12.97	12.07	12.03	11.63	11.80
	HSD	abc	abc	a*	ab	abc	abc	c	bc
**Acidity**	Mean	1.24	1.23	1.27	1.20	1.18	1.14	1.16	1.17
	HSD	a	a	a	a	a	a	a	a
**Ripeness**	Mean	10.11	10.30	10.47	10.84	10.24	10.59	10.16	10.12
	HSD	a	a	a	a	a	a	a	a
**Vitamin C**	Mean	31.45	29.29	32.72	32.07	31.36	32.67	31.21	30.36
	HSD	a	a	a	a	a	a	a	a

Table note:

(*) emphasizes when there is a statistically significant difference (alpha ≤ 0.05) between a treatment and C+. The Kruskal-Wallis test (KW) was used when residuals did not meet normality or variances did not meet the homoscedasticity requirement, while ANOVA Tukey *post hoc* tests (HSD) were applied when data requirements were met. Units are: diameter (mm), weight (g), CI (CI value), % rind, juice and pulp (percentages), pH (pH 0 to 14), soluble solids (º Brix), total acidity (per-centage), ripeness (maturity index, MI) and vitamin C (mg/100 g juice).

### Phytotoxic side effects of treatments

The ammonium nitrate treatment caused moderate tree defoliation 4 weeks after the last application. Apart from this side effect, no chlorosis, necrosis or defoliation was observed in the other treatments 2 and 4 weeks after the last application.

## Discussion

### Seed reduction by treatments

The positive control produced an average of 3.3 seeds per fruit, while the negative control (anti-insect nets) obtained 0.07 seeds per fruit. These results agree with previous studies carried out on the Nadorcott variety. Open-pollination Nadorcott trees usually present an average of around 2 to 4 seeds per fruit and between 85–100% of fruit with seeds [17, 18, 30]. The usual average seed number in net-covered Nadorcott trees is around 0.03–0.07 seeds per fruit and between 3–7% of fruit with seeds [14, 17, 18]. In a field experiment conducted under low cross-pollination conditions, the Nadorcott positive control produced an average of 0.99 seeds per fruit [12]. Therefore, the negative and positive controls in this study showed the usual values under suitable cross-pollination conditions, which allowed an adequate evaluation of treatments.

The elemental sulfur treatment was the only one (except for the negative control) to bring about a statistically significant reduction in seed number per fruit compared to the positive control. The other treatments did not significantly reduce seed number per fruit. This result completely agrees with a previous study, which evaluated the effect of the same products on pollen tube growth [13]. In that case, isolated flowers were treated and the sulfur treatment inhibited pollen tube growth by 94–100%. The saccharose treatment led to the most pollen tube growth inside Nadorcott flower stigmas [13]. When products were applied to whole trees in the present study, the sulfur treatment (S_8_) reduced seed number per fruit by 87%. Compared to the laboratory results [13], this loss of effectiveness could be because the product did not reach all the flowers upon anthesis. Citrus flowering is a long process that takes more than 4 weeks [31, 32] and it can, thus, be difficult to reach all the flowers at the right time. According to the pollen tube growth study, the saccharose treatment brought about the biggest seed number.

The frequency of seedy mandarins fell from 88.3% in C+ to 20.8% in the sulfur treatment, which meant that the sulfur treatment prevented 100% fertilizations in many flowers. When seed reduction was analyzed only in the seedy mandarins, the sulfur treatment continued to reduce seed number (S5 File). This revealed a partial effect of sulfur on some flowers. This partial effect was probably because: (1) elemental sulfur did not reach the entire stigma surface uniformly (due to the position of some flowers on trees); or (2) the product arrived late once pollination and pollen tube growth had begun and, therefore, inhibited part of fertilizations, but not them all. Citrus flowers open every day during the flowering period [31, 32], and treatments were carried out at three specific times (3 applications 7 days apart). It is likely that some flowers might have opened and pollinated shortly after one application and, thus, the effect of the next application was partial.

Never before has a product shown such good effectiveness in reducing seed numbers in Nadorcott mandarin. The most recent studies indicated effectiveness at around 35%, but this seed reduction is not sufficient for commercial purposes [12, 17, 18]. None of the products proposed to date (gibberellic acid, copper sulfate, *Capsicum annuum* extract, zinc, etc.) have shown good effectiveness when applied to whole trees under field conditions [12]. In addition, copper sulfate, which has been initially used by farmers, was tested in an oral and contact exposure bioassay, proved toxic and compromised bee workers’ survival [33]. Therefore, it is unsuitable for agronomic use. Today none of these treatments is currently being used by citrus growers.

### Effect on yield

The influence of treatments on yield was not easy to assess given the wide variability among trees, even within treatments. This variability could be accentuated by trees’ young age (only 9 years old) and Nadorcott’s alternate-bearing behavior. Young trees are less resilient than adult trees and are unable to recover fruit set under stressful conditions. Therefore, minor disturbances can markedly affect the yields of young trees [34–36]. In addition, alternate bearing is common in late citrus [37], and Nadorcott mandarin trees can present severe alternate bearing with some erratic behavior [38].

Despite these limitations, the net-covered trees obtained the lowest yields, which agrees with previous studies [12, 14, 17, 18]. The magnitude of this reduction caused by nets has been discussed by several authors, and ranges from 66% [18] to no reduction [39], although reductions of around 20% are common [12, 14, 17]. The sulfur treatment did not show any statistically significant differences compared to C+, but a yield reduction trend was observed. Therefore, future studies need to make more sampling efforts using more data collected from adult trees.

### Effect on fruit quality and phytotoxicity

The influence of treatments on fruit quality (except for seed number) was mild or null. Treatments were applied during flowering in the flower state (April 2017). Fruit reached maturity 10 months later (February 2018), which may lie behind this poor influence on these variables. The sulfur treatment did not show any statistically significant differences in % juice, % rind, % pulp, fruit weight, juice volume, pH, total acidity, vitamin C, MI, ºBrix and CI compared to the positive control. The obtained values were the usual quality values for citrus fruit [40, 41].

The negative control and the ammonium nitrate treatment showed a smaller fruit diameter with statistically significant differences. The ammonium nitrate treatment caused moderate defoliation. The detrimental effect of ammonium nitrate on leaves most likely affected tree nutritional status of the tree, causing smaller fruit. Finally, both seed number and fruit diameter obtained the lowest values in the negative control. The relation between seed number and fruit size has been widely studied in citrus and many other fruit [35, 42–44]. Studies suggest that seeds stimulate fruit growth via gibberellic acid production [44, 45]. Therefore, the size reduction observed in the C- and seedless fruit is in accordance with previous studies.

### Importance and future research

The results reported herein open up new possibilities to achieve an effective agronomic treatment to reduce seed number in traditional seedy mandarins. To fulfill this objective, a better understanding of the mechanism by which sulfur blocks pollen tube growth inside stigmas is necessary. In addition, experiments run under semireal field conditions with tractor applications to entire rows of adult trees and different doses should be tested. Applying the sulfur treatment to other mandarins (not only Nadorcott), or even to other citrus fruit like oranges or lemons, should also be explored to determine if it can be useful with other crops.

## Conclusions

Of the six products (ammonium nitrate, potassium nitrate, sulfur, saccharose, methylcellulose, callose) tested in the field for seed reduction in Nadorcott mandarin, only sulfur showed good effectiveness (87% seed reduction). No statistically significant differences in yield or fruit quality were observed between the positive control and the sulfur treatment. Therefore, sulfur is a promising candidate for developing treatments to prevent seeds in fruit. No product previously tested under field conditions has obtained such excellent effectiveness.

Net-covered trees, which is a technique used by some farmers, showed the smallest seed number per fruit, but also the lowest yield and the smallest fruit diameter. The saccharose treatment had the opposite effect to that of sulfur because it increased seed number per fruit, but with no statistically significant differences compared to C+. All these findings can help to develop new techniques, although more studies are needed to test, for example, different treatment doses or the effect on other mandarin or citrus crop varieties.

## Supporting information

S1 Fig The experimental plot with six blocks and the distribution of the treated trees. Each white square is a tree. Treatments are denoted as: 1 (red) sulfur; 2 (yellow) ammonium nitrate; 3 (green) potassium nitrate; 4 (light blue) saccharose; 5 (orange) methyl cellulose; 6 (violet) callose; 7 (light green) negative control; 8 (dark blue) positive control.
(TIF)

S1 File Comprehensive data and statistical analysis. Zip file with the data and R scripts for the statistical analysis.
(ZIP)

S2 File Effect of blocks on seed number per fruit. Violin plot of the seed number per fruit for each block with the Kruskal-Wallis test.
(PDF)

S3 File Comparison between the elemental sulfur and the positive control. Density plot of seed number per fruit for the sulfur treatment (yellow) and the positive control (blue), with an indication of the mean in each one (vertical dotted lines).
(PDF)

S4 File Comparison between the elemental sulfur and the negative control. Density plot of seed number per fruit for the sulfur treatment (yellow) and the negative control (blue), with an indication of the mean in each one (vertical dotted lines).
(PDF)

S5 File Effect of treatments on seed number per fruit ONLY with seedy fruit. The table and figure of the effect of treatments on seed number per fruit in the seedy fruit per treatment.
(PDF)

S6 File Effect of blocks on yield. Violin plot of the yield for each block with the ANOVA Tukey *post hoc* test (HSD).
(PDF)

S7 File Relation between presence of seeds and fruit size. Box plot for the effect of presence of seeds on fruit diameter with the Kruskal-Wallis test.
(PDF)
