# Supplementary figures and images for: Agronomic treatments to avoid presence of seeds in Nadorcott mandarin II. Effect on seed number per fruit and yield
Source: PLoS One. 2022 Dec 9;17(12):e0278934. doi: 10.1371/journal.pone.0278934 (PMC9733848; doi:10.1371/journal.pone.0278934)

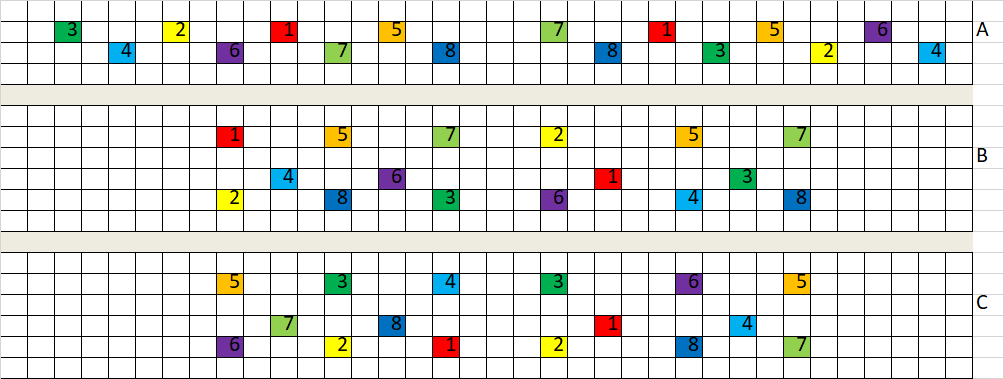

Supplement: S1 Fig — Each white square is a tree. Treatments are denoted as: 1 (red) sulfur; 2 (yellow) ammonium nitrate; 3 (green) potassium nitrate; 4 (light blue) saccharose; 5 (orange) methyl cellulose; 6 (violet) callose; 7 (light green) negative control; 8 (dark blue) positive control. (TIF) [file pone.0278934.s001.tif]
