# Supplementary material for: Agronomic treatments to avoid presence of seeds in Nadorcott mandarin II. Effect on seed number per fruit and yield
Source: PLoS One. 2022 Dec 9;17(12):e0278934. doi: 10.1371/journal.pone.0278934 (PMC9733848; doi:10.1371/journal.pone.0278934)
Supplement: S2 File — Violin plot of the seed number per fruit for each block with the Kruskal-Wallis test. (PDF) [file pone.0278934.s003.pdf]

Number of seeds in Nadorcott

Effect of treatments

2022-09-09

Contents

Block effect 1
Treatment effect 2
Densities 4

Block effect

There is no differences between blocks.

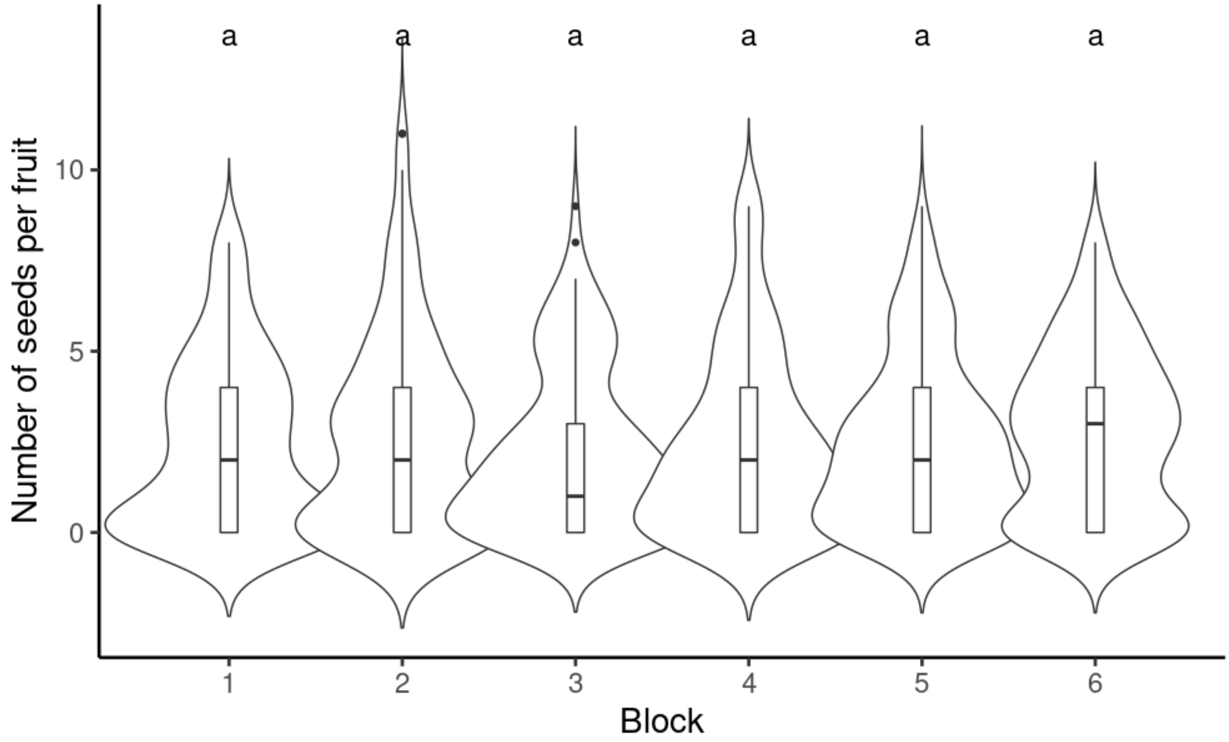

Figure 1: Figure 1: Violin plot of seeds for each block. Different letters represent significant differences in Kruskal-Wallis test (KW), for alpha = 0.05.

Treatment effect

Nemenyi's non-parametric all-pairs comparison test for Kruskal-type ranked data using Tukey distribution to determine the p-values. Package PMCMRplus: Pohlert T (2022). *PMCMRplus: Calculate Pairwise Multiple Comparisons of Mean Rank Sums Extended*. R package version 1.9.6, https://CRAN.R-project.org/package=PMCMRplus.

```
##          C-      Sulfur  A_Nitrat K_Nitrat Sacchar M_Cellul Callose
## Sulfur   0.80    -       -        -        -       -        -
## A_Nitrat 8.0e-14 9.1e-14 -        -        -       -        -
## K_Nitrat < 2e-16 8.2e-14 0.95     -        -       -        -
## Sacchar  < 2e-16 5.5e-14 0.84     1.00     -       -        -
## M_Cellul 5.9e-14 6.8e-14 1.00     0.92     0.79    -        -
## Callose  < 2e-16 6.2e-14 1.00     1.00     0.98    1.00     -
## C+       < 2e-16 7.9e-14 0.98     1.00     1.00    0.97     1.00
```

Table 1: Table 1: Effect of treatment on seeds. Kruskal-Wallis posthoc test (KW) letters should be used because residuals do not meet normality requirement for Anova: Shapiro.p = 4.98e-17 . Holm's correction method (Holm 1979) was used.. Different letters mean significant differences for alpha = 0.05. q stands for the studentized range in the Tukey test (HSD).

treatment	N	Median	Mean	sd	se	skew	kurtosis	Shapiro	HSD	KW
C-	120	0	0.07	0.25	0.02	3.52	10.56	0	b	b
Sulfur	120	0	0.41	0.98	0.09	3.09	11.07	0	b	b
A_Nitrat	105	3	2.88	2.08	0.20	0.68	0.38	0	a	a
K_Nitrat	120	3	3.33	2.27	0.21	0.34	-0.81	0	a	a
Sacchar	120	3	3.42	2.27	0.21	0.52	-0.30	0	a	a
M_Cellul	120	3	2.91	2.20	0.20	0.79	0.61	0	a	a
Callose	120	3	3.18	2.50	0.23	0.90	0.22	0	a	a
C+	120	3	3.30	2.35	0.21	0.53	-0.36	0	a	a

	Df	Sum Sq	Mean Sq	F value	Pr(>F)	q	eta.sq	Levene	Shapiro
treatment	7	1584.711	226.387	56.133	0	4.296	0.295	0	NA
Residuals	937	3778.999	4.033	NA	NA	NA	NA	NA	0

Of course the groups cannot meet the normality (Shapiro), or the homoskedasticity (Levene) criteria, because the groups without seeds have very low variability. Levene p = $2.0275606 \times 10^{-51}$. Shapiro p = $4.9771027 \times 10^{-17}$

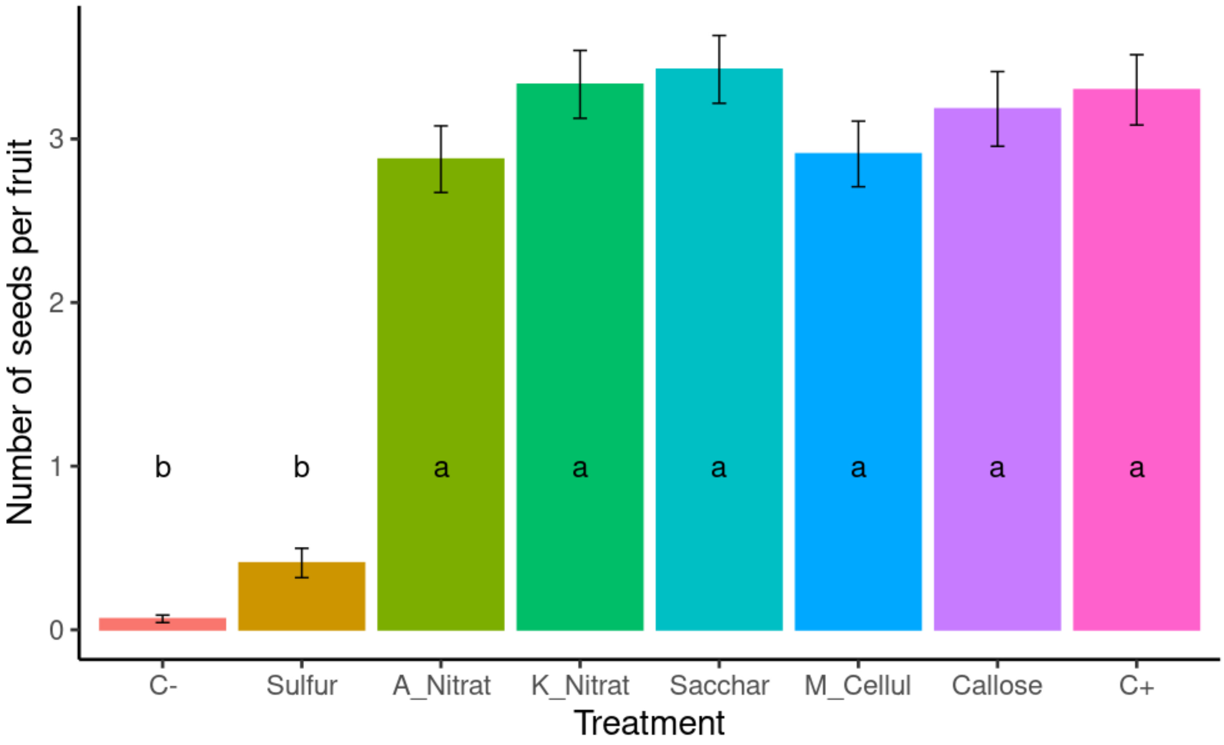

Figure 2: Figure 2: Barplot for the effect of treatment on seeds. Error bars correspond with standard error. Different letters mean significant differences in Kruskal-Wallis posthoc test (KW), for alpha = 0.05.
