# Supplementary material for: Agronomic treatments to avoid presence of seeds in Nadorcott mandarin II. Effect on seed number per fruit and yield
Source: PLoS One. 2022 Dec 9;17(12):e0278934. doi: 10.1371/journal.pone.0278934 (PMC9733848; doi:10.1371/journal.pone.0278934)
Supplement: S4 File — Density plot of seed number per fruit for the sulfur treatment (yellow) and the negative control (blue), with an indication of the mean in each one (vertical dotted lines). (PDF) [file pone.0278934.s005.pdf]

Comparison between elemental sulfur treatment and negative control.

Mann-Whitney-Wilcoxon Test, 0.41 seeds per fruit sulfur vs. 0.07 seeds per fruit negative control (C-), p- value Whitney U test = 8.65112610^{-4}.

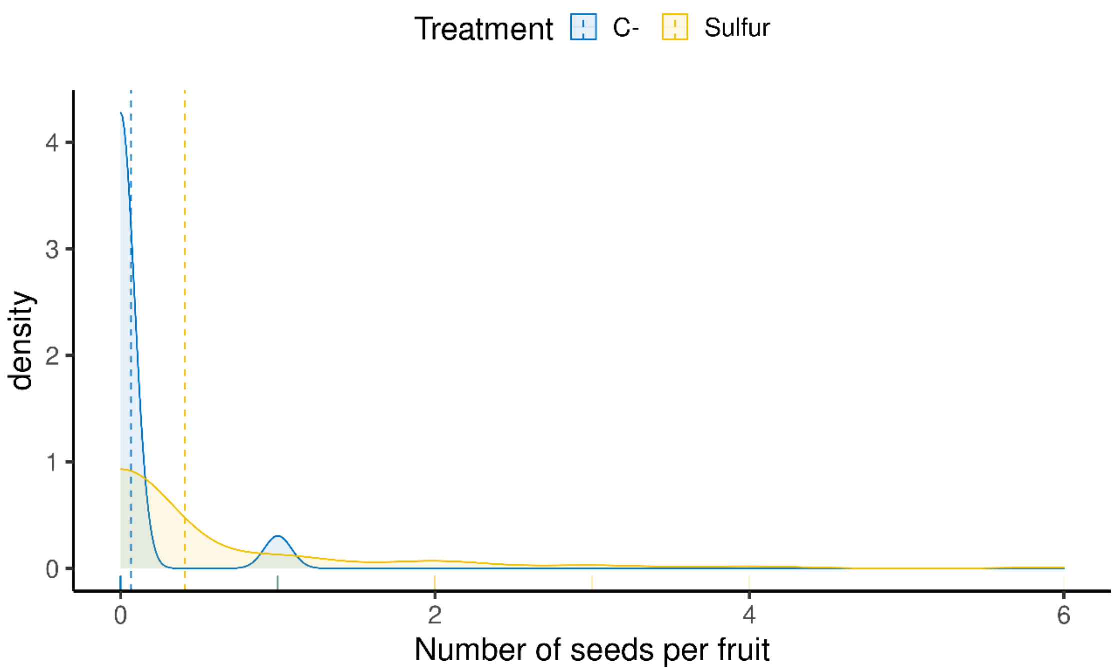

Fig. Density plot of the number of seeds per fruit for the sulfur treatment (yellow) and the negative control (blue), with an indication of the mean in each of them (vertical dotted lines).
