# Supplementary material for: Agronomic treatments to avoid presence of seeds in Nadorcott mandarin II. Effect on seed number per fruit and yield
Source: PLoS One. 2022 Dec 9;17(12):e0278934. doi: 10.1371/journal.pone.0278934 (PMC9733848; doi:10.1371/journal.pone.0278934)
Supplement: S5 File — The table and figure of the effect of treatments on seed number per fruit in the seedy fruit per treatment. (PDF) [file pone.0278934.s006.pdf]

Effect of treatments on seed number per fruit ONLY with seedy fruits.

Table. Effect of the treatments on the number of seeds in the fruits with seeds (excluding those that do not have seeds). The Kruskal Wallis test for comparison of means was used due to the non-normality of the residuals.

Treatment	N	mean	KW	sd	se	skew	kurtosis	Shapiro
C-	8	1	b	0	0	NaN	NaN	-
Sulfur	25	1,96	b	1,27	0,25	1,66	2,98	5,48E-05
A_Nitrat	92	3,28	a	1,90	0,20	0,87	0,81	1,05E-05
K_Nitrat	107	3,74	a	2,07	0,20	0,43	-0,81	1,74E-05
Sacchar	109	3,77	a	2,09	0,20	0,68	-0,21	1,08E-05
M_Cellul	104	3,36	a	2,01	0,20	0,97	1,05	1,38E-06
Callose	108	3,54	a	2,39	0,23	0,97	0,26	1,14E-07
C+	106	3,74	a	2,15	0,21	0,65	-0,25	2,37E-05

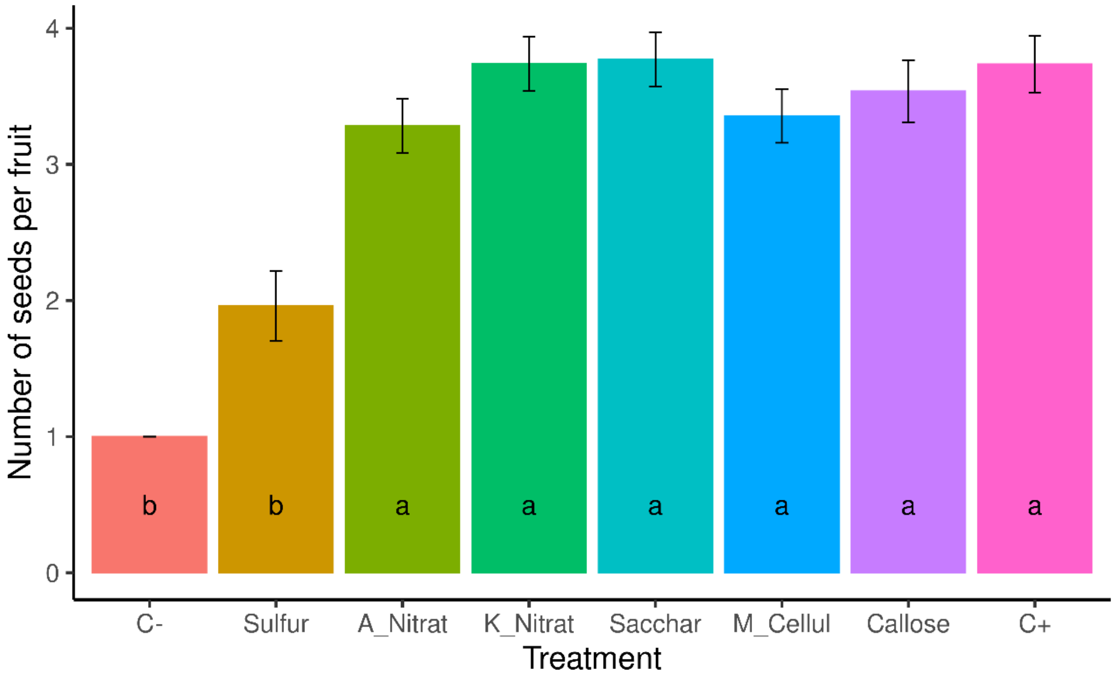

Fig. Barplot of the number of seeds per fruit, in the fruits with seeds, for each treatment. Different letters mean significant differences in Kruskal-Wallis posthoc test (KW), for alpha = 0.05

It is clearly seen that there is still a strong difference between sulfur treatment and the rest of the treatments.
