# Supplementary material for: Agronomic treatments to avoid presence of seeds in Nadorcott mandarin II. Effect on seed number per fruit and yield
Source: PLoS One. 2022 Dec 9;17(12):e0278934. doi: 10.1371/journal.pone.0278934 (PMC9733848; doi:10.1371/journal.pone.0278934)
Supplement: S6 File — Violin plot of the yield for each block with the ANOVA Tukey post hoc test (HSD). (PDF) [file pone.0278934.s007.pdf]

Yield in Nadorcott

For article

2022-09-09

Contents

Block effect 1
Yield 2
Densities 4
. 6

Block effect

There is a small difference between block 2 (more yield) and block 5 (less yield).

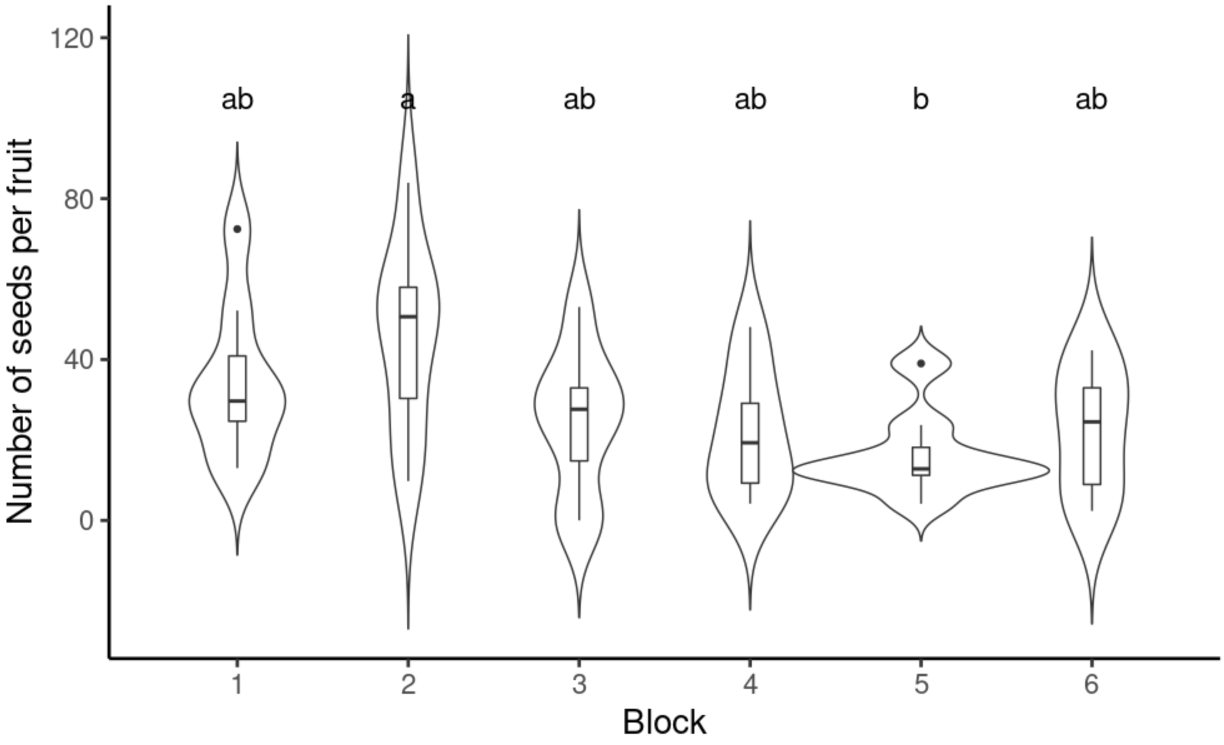

Figure 1: Figure 1: Violin plot of yield for each block. Different letters represent significant differences in Anova Tukey posthoc test (HSD), for alpha = 0.05.

Yield

```
## Analysis of Variance Table
##
## Response: yield
##           Df Sum Sq Mean Sq F value   Pr(>F)
## treatment  7 5503.3  786.19  3.5968 0.005084 **
## block      5 4614.5  922.89  4.2222 0.004156 **
## Residuals 35 7650.3  218.58
## ---
## Signif. codes:  0 '***' 0.001 '**' 0.01 '*' 0.05 '.' 0.1 ' ' 1
```

Table 1: Table 1: Effect of treatments on yield, taking into account the block effect. Anova Tukey posthoc test (HSD) letters should be used because residuals meet normality requireement for Anova: Shapiro.p = 0.0511. HSD letters were calculated taking block effect into account. Different letters mean significant differences for alpha = 0.05. q stands for the studentized range in the Tukey test (HSD). 'Fr' stands for Friedman test.

treatment	N	Median	Mean	sd	se	Shapiro	HSD	Fr
C-	6	8.57	11.36	12.17	4.97	0.13	b	b
Sulfur	6	11.38	17.20	13.70	5.59	0.23	ab	ab
A_Nitrat	6	15.52	14.59	10.61	4.33	0.89	ab	ab
K_Nitrat	6	29.40	33.46	26.64	10.88	0.06	ab	ab
Sacchar	6	38.64	40.51	11.66	4.76	0.64	a	a
M_Cellul	6	33.27	38.86	22.73	9.28	0.76	a	ab
Callose	6	33.67	33.88	16.93	6.91	0.31	ab	ab
C+	6	27.32	30.58	18.86	7.70	0.16	ab	ab

	Df	Sum Sq	Mean Sq	F value	Pr(>F)	q	eta.sq	Levene	Shapiro
treatment	7	5503.303	786.186	2.564	0.028	4.521	0.31	0.391	NA
Residuals	40	12264.768	306.619	NA	NA	NA	NA	NA	0.051

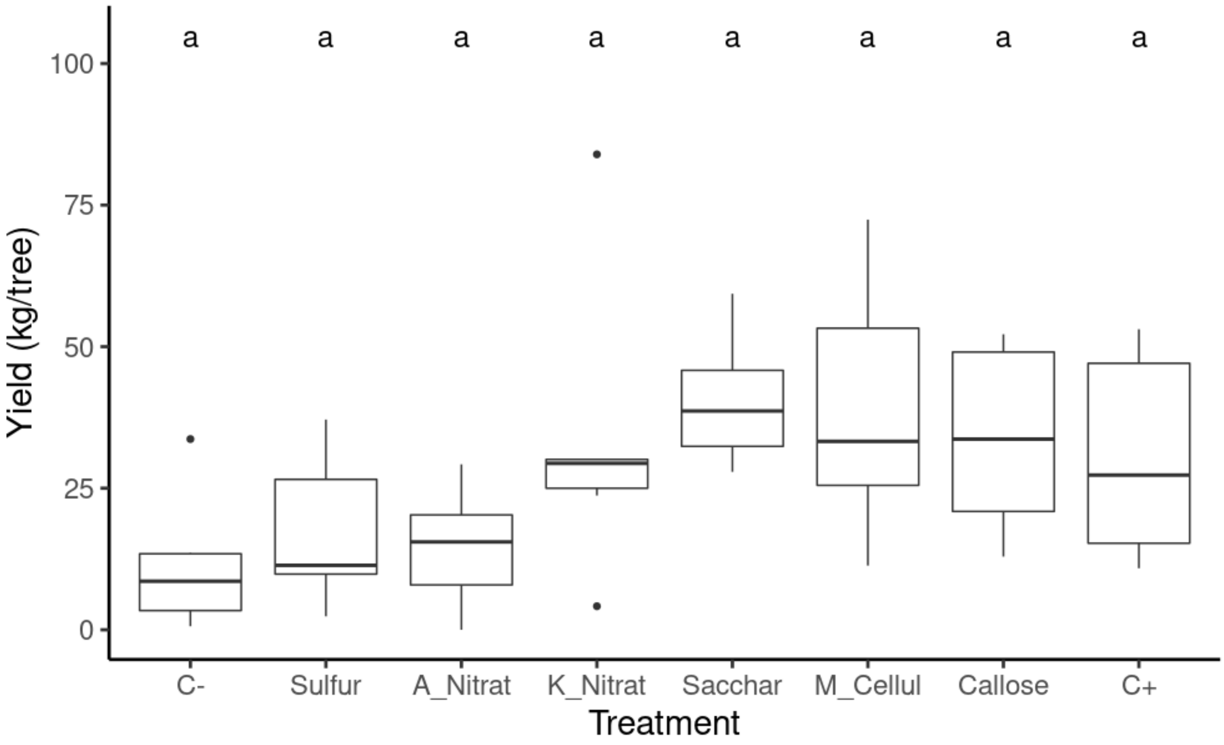

Figure 2: Figure 1: Boxplot of yield for each treatment. Different letters represent significant differences inAnova Tukey posthoc test (HSD), for alpha = 0.05.

Densities

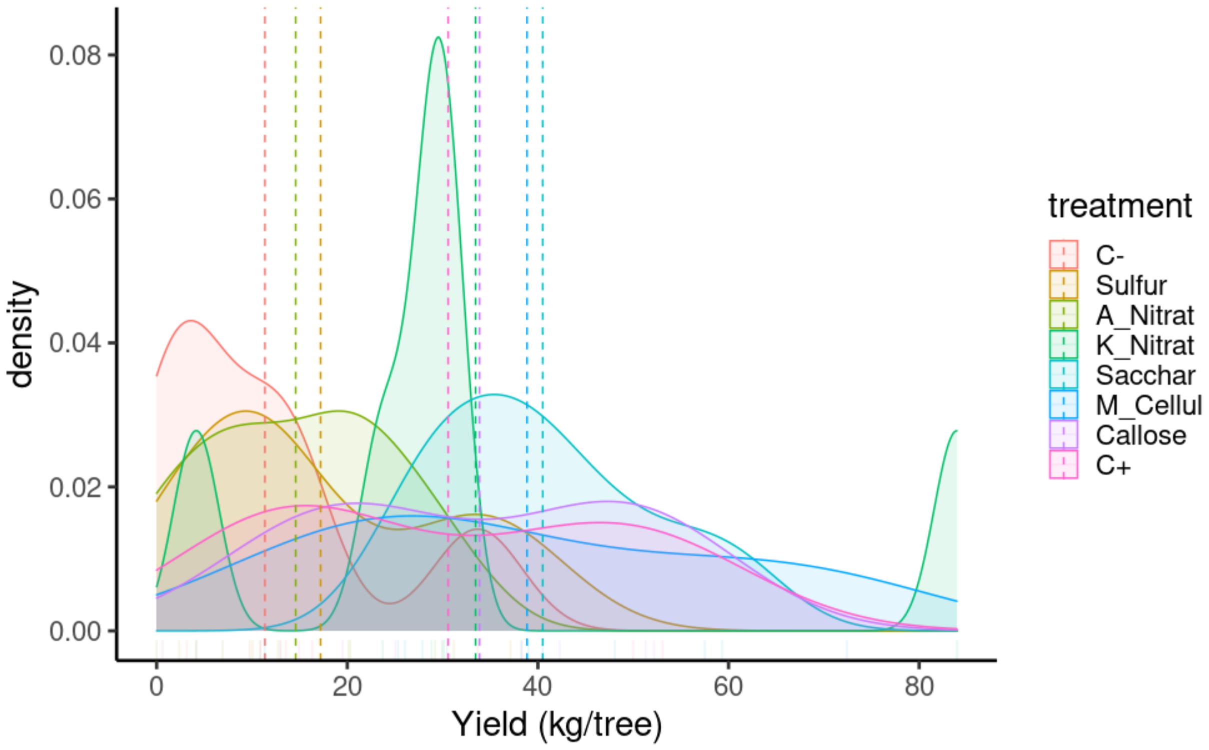

Figure 3: Figure 2: Densities for yield according to treatment. Dotted lines represent the mean value.

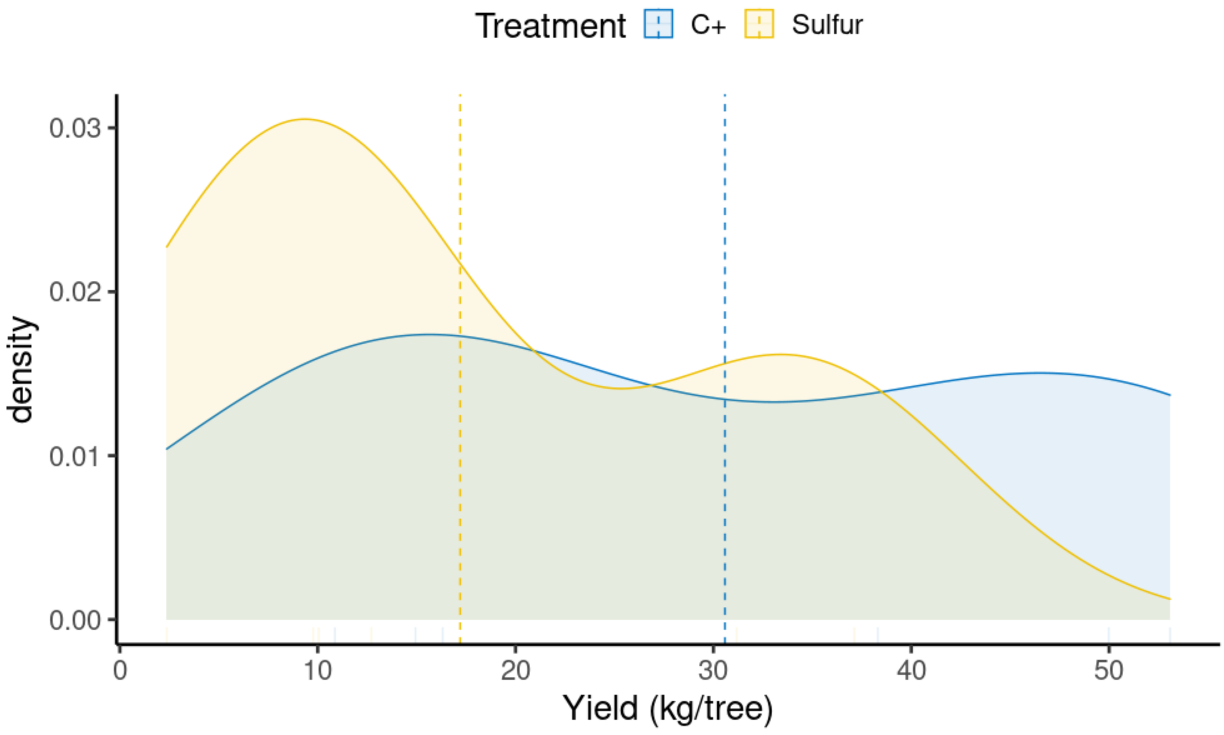

Figure 4: Figure 3: Densities for yield comparing C+ with Sulfur. Dotted lines represent the mean value.

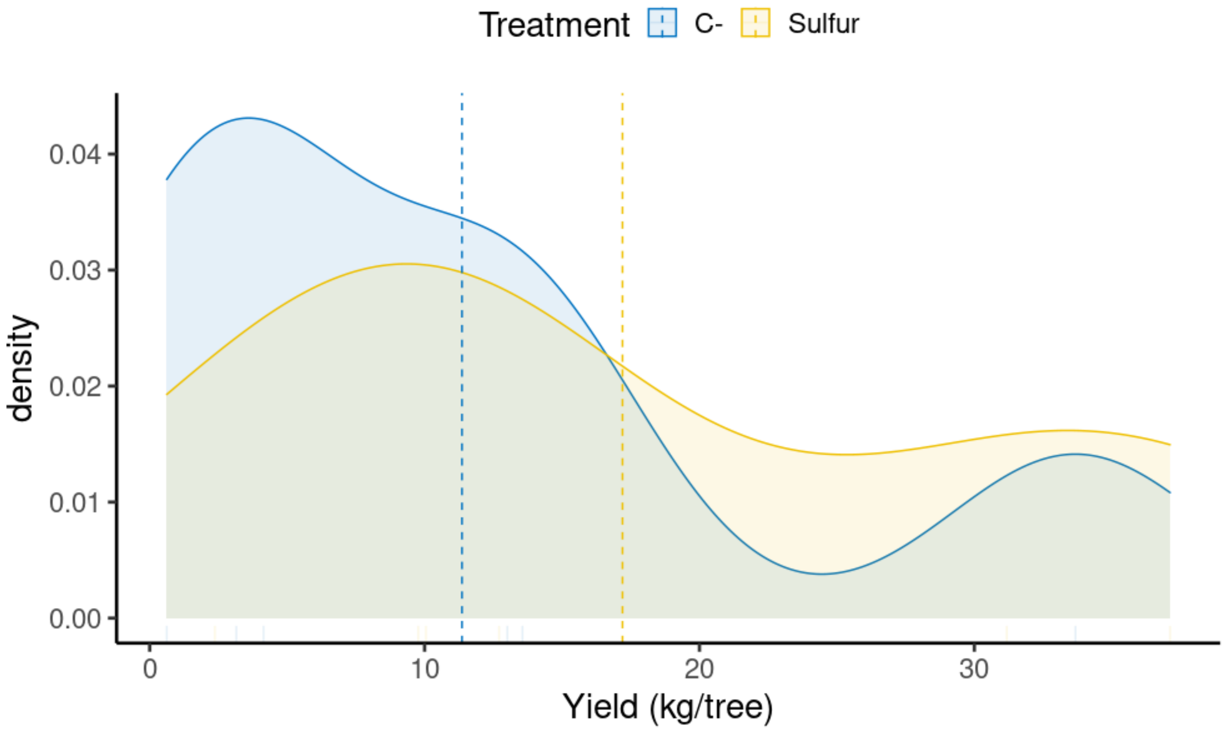

Figure 5: Figure 4: Densities for yield comparing C- with Sulfur. Dotted lines represent the mean value.

Reproducibility

Written in Rmarkdown, using Rstudio.

- System: R version 4.2.1 (2022-06-23) x86_64-pc-linux-gnu (64-bit) Ubuntu 22.04.1 LTS
- Base packages: stats 4.2.1, graphics 4.2.1, grDevices 4.2.1, utils 4.2.1, datasets 4.2.1, methods 4.2.1, base 4.2.1.
- Other loaded packages: car 3.0.13, carData 3.0.5, bibtex 0.4.2.3, agricolae 1.3.5, ggpubr 0.4.0, ggsci 2.9, ggplot2 3.3.6, dplyr 1.0.9, tidyr 1.2.0, readxl 1.4.0, knitr 1.39.
