# Supplementary material for: Agronomic treatments to avoid presence of seeds in Nadorcott mandarin II. Effect on seed number per fruit and yield
Source: PLoS One. 2022 Dec 9;17(12):e0278934. doi: 10.1371/journal.pone.0278934 (PMC9733848; doi:10.1371/journal.pone.0278934)
Supplement: S7 File — Box plot for the effect of presence of seeds on fruit diameter with the Kruskal-Wallis test. (PDF) [file pone.0278934.s008.pdf]

Size of fruits by presence of seeds

2022-09-09

Contents

Frequencies of fruits with seeds or seedless 1
Relationship between the presence of seeds and size of the fruit 2

Frequencies of fruits with seeds or seedless

Table 1: Table 1: Number of fruits with seeds and seedless for each treatment.

	C-	Sulfur	A_Nitrat	K_Nitrat	Sacchar	M_Cellul	Callose	C+
seedless	112	95	13	13	11	16	12	14
with_seeds	8	25	92	107	109	104	108	106

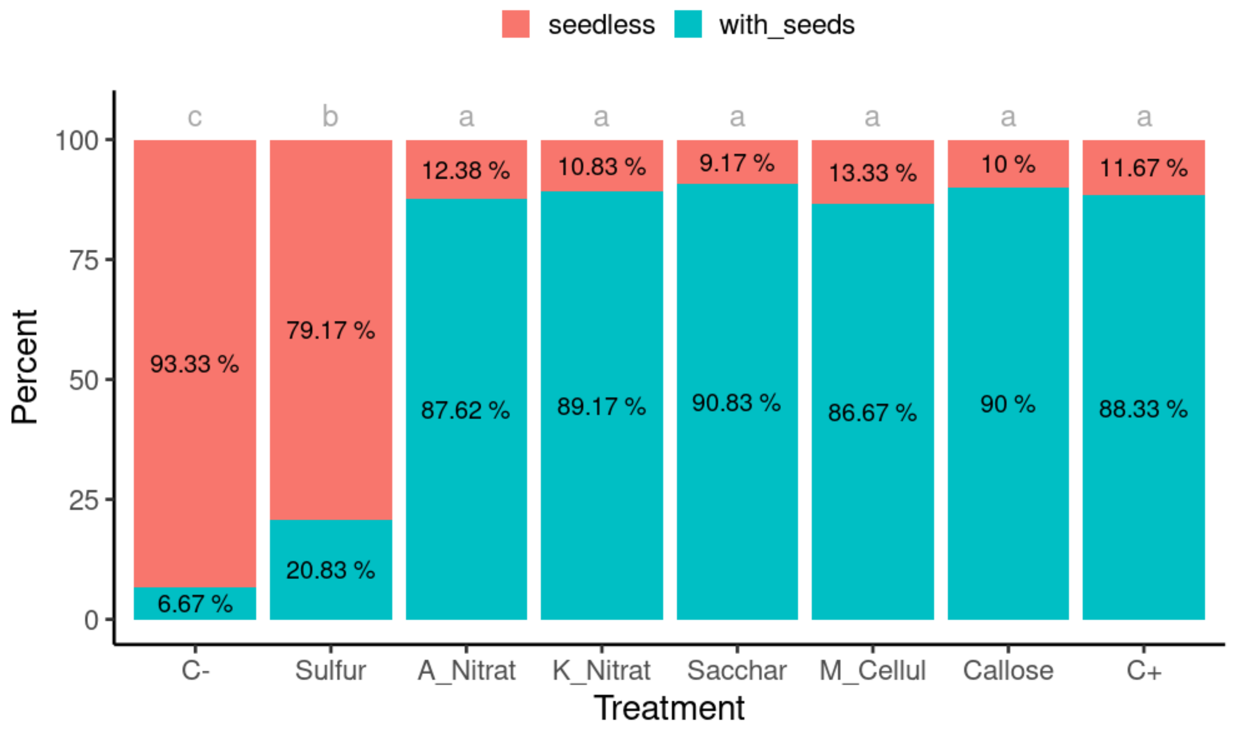

Figure 1: Figure 1: Percents of seed.presence on treatment. For post-hoc analyses was used the Fisher's Exact Test for Count Data with simulated p-value (based on 2000 replicates) with Holm's correction method (Holm 1979). Total Fisher test p = 5e-04.

Relationship between the presence of seeds and size of the fruit

The same fruits were measured (diameter in mm) before cutting them to count the seeds.

Table 2: Table 2: Effect of seed.presence on diameter. Kruskal-Wallis posthoc test (KW) letters should be used because residuals do not meet normality requirement for Anova: Shapiro.p = 1.01e-06 . Holm's correction method (Holm 1979) was used.. Different letters mean significant differences for alpha = 0.05. q stands for the studentized range in the Tukey test (HSD).

seed.presence	N	Median	Mean	sd	se	skew	kurtosis	Shapiro	HSD	KW
seedless	286	47	47.02	3.88	0.23	-0.06	0.67	0	b	b
with_seeds	659	50	49.48	3.88	0.15	-0.15	0.65	0	a	a

	Df	Sum Sq	Mean Sq	F value	Pr(>F)	q	eta.sq	Levene	Shapiro
seed.presence	1	1207.456	1207.456	80.257	0	2.775	0.078	0.829	NA
Residuals	943	14187.344	15.045	NA	NA	NA	NA	NA	0

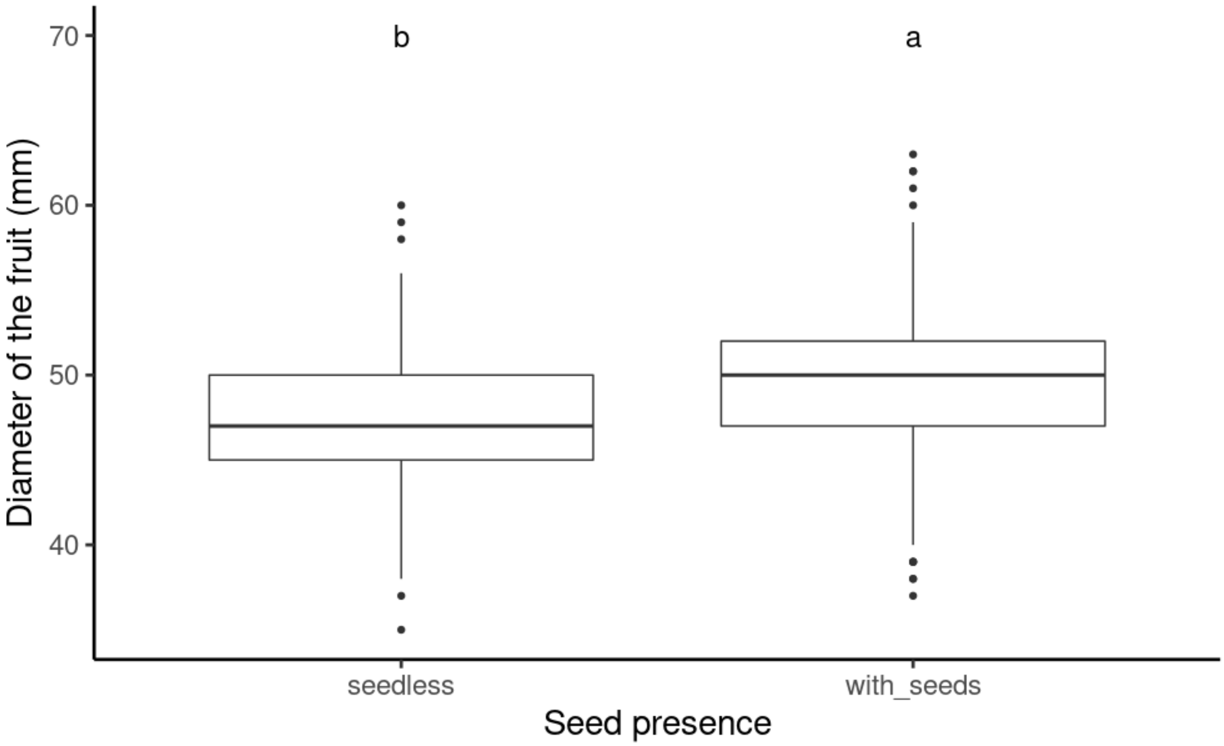

Figure 2: Figure 2: Boxplot for the effect of the presence of seeds on the diameter of the fruit. Different letters mean significant differences in Kruskal-Wallis posthoc test (KW), for alpha = 0.05.

Fruits with seeds are about 3mm larger than seedless fruits.

Reproducibility

Written in Rmarkdown, using Rstudio.

- System: R version 4.2.1 (2022-06-23) x86_64-pc-linux-gnu (64-bit) Ubuntu 22.04.1 LTS
- Base packages: stats 4.2.1, graphics 4.2.1, grDevices 4.2.1, utils 4.2.1, datasets 4.2.1, methods 4.2.1, base 4.2.1.
- Other loaded packages: car 3.0.13, carData 3.0.5, bibtex 0.4.2.3, agricolae 1.3.5, ggpubr 0.4.0, ggsci 2.9, ggplot2 3.3.6, dplyr 1.0.9, tidyr 1.2.0, readxl 1.4.0, knitr 1.39.
